# Changes in HER2 status and survival outcomes in patients with non-pathological complete response after neoadjuvant targeted treatment

**DOI:** 10.1097/MD.0000000000034903

**Published:** 2023-09-29

**Authors:** Xiaofei Ren, Xiangmei Zhang, Xiangmin Ma, Chao Yang, Jingping Li, Beichen Liu, Chao Shi, Yunjiang Liu

**Affiliations:** a Hebei Provincial Key Laboratory of Tumor Microenvironment and Drug Resistance, Hebei Medical University, Shijiazhuang City, Hebei, China; b Department of Breast Center, Fourth Hospital of Hebei Medical University, Shijiazhuang City, Hebei, China; c Department of Hematology, Fourth Hospital of Hebei Medical University, Shijiazhuang City, Hebei, China; d Research Center, Fourth Hospital of Hebei Medical University, Shijiazhuang City, Hebei, China; e Department of Breast Surgery, Handan Central Hospital, Handan City, Hebei, China.

**Keywords:** breast cancer, human epidermal growth factor receptor 2, neoadjuvant treatment, pathological complete response, targeted treatment

## Abstract

To study the changes in human epidermal growth factor receptor 2 (HER2) expression in patients with HER2-positive breast cancer before and after neoadjuvant treatment. The clinicopathologic data of 499 patients with HER2-positive breast cancer who completed neoadjuvant treatment and surgery at the Fourth Hospital of Hebei Medical University from 2018 to 2021 were retrospectively analyzed. According to the new adjuvant regimen, 298 patients were divided into the trastuzumab + pertuzumab combined chemotherapy group (dual target group), and 201 patients were divided into the trastuzumab combined chemotherapy group (single target group).The effect of different neoadjuvant regimens on HER2 status was analyzed by comparing HER2 expression before and after treatment. A total of 255 of 499 neoadjuvant patients with HER2-positive breast cancer achieved a pathological complete response (pCR). pCR was achieved in 60.07% (179/298) of the dual target group and 37.81% (76/201) of the single target group, and the difference was statistically significant (*χ*² = 23.795, *P* < .001). Among 244 cases of HER2-positive breast cancer that did not reach pCR (non-pCR), there was a certain negative conversion rate of HER2 expression after neoadjuvant treatment, and the overall negative conversion rate was 13.11% (32/244). The negative conversion rates of the dual target group was 17.65% (21/119) and single target group was 8.80% (11/125), (*χ*² = 4.188, *P* = .041). The DFS of 499 patients in the pCR group was 98.43% (251/255), which was significantly higher than that in the non-pCR group 92.21% (225/244), (*χ*² = 8.536, *P* = .003). Only 2 (0.20%) of 32 patients with negative HER2 had recurrence and metastasis. Neoadjuvant treatment had an effect on the expression status of HER2, especially in the dual target group. For patients with negative HER2, the optimal treatment strategy remains to be explored, but continued anti-HER2 treatment is still recommended.

## 1. Introduction

According to the latest data on the 2020 global burden of cancer, breast cancer has surpassed lung cancer to become the most common malignant tumor.^[1]^ Human epidermal growth factor receptor 2 (HER2)-positive breast cancers account for 20% to 30% of all breast cancers, with biological characteristics such as a high degree of malignancy, insensitivity to traditional chemotherapy and poor clinical prognosis.^[2,3]^ Trastuzumab, a humanized monoclonal antibody that specifically targets HER2, can significantly improve the prognosis of HER2-positive breast cancer.^[4]^ Neoadjuvant chemotherapy is the standard treatment for locally advanced breast cancer. On the one hand, it can achieve the effect of local control, downstaging surgery or tumor shrinkage to achieve breast conservation. On the other hand, it can be used as in vivo drug sensitivity test to guide subsequent treatment and improve prognosis and long-term survival.^[4]^ For patients with HER2-positive breast cancer, neoadjuvant chemotherapy combined with targeted treatment is superior to chemotherapy alone, and improves the pathologic complete response (pCR) of the breast and lymph nodes.^[5]^ However, for non-pCR patients, the probability of recurrence and metastasis in a short period of time is significantly increased, and intensive treatment after neoadjuvant and surgical treatment has important clinical significance. At present, there is a certain negative conversion rate of HER2 in non-pCR patients after neoadjuvant treatment with trastuzumab, and it is controversial whether postoperative adjuvant treatment should be intensified for the negative conversion population.^[6]^ The aim of this study was to analyze the clinicopathological data of 499 patients with HER2-positive breast cancer and to investigate the changes in HER2 expression status in patients with HER2-positive breast cancer after neoadjuvant treatment.

## 2. Patients and methods

### 2.1. Study design and patients

The clinicopathological data of breast cancer patients who completed neoadjuvant treatment and surgical treatment in the Breast Center of the Fourth Hospital of Hebei Medical University from 2018 to 2021 were collected. The inclusion criteria were as follows: pathological examination confirmed breast cancer before neoadjuvant treatment, HER2 (+++) was detected by immunohistochemistry before neoadjuvant treatment, HER2 (++) was detected by fluorescence in situ hybridization (FISH), no distant metastasis, and completion of the scheduled 6 to 8 cycles of neoadjuvant chemotherapy. Exclusion criteria were as follows: previous history of other malignant tumors, previous history of endocrine treatment, targeted treatment and radiotherapy, and other immunohistochemical subtypes. A total of 499 patients met the inclusion criteria. All patients were female, aged from 23 to 70 years, with a median age of 51 years. According to the 2017 American Joint Committee on Cancer clinical staging criteria. According to the neoadjuvant regimen, patients were divided into a dual target group (298 cases) and a single-target group (201 cases). The drug usage and dosage were determined according to the 2017 Chinese Society of Clinical Oncology breast cancer diagnosis and treatment guidelines.

HER2 testing was performed according to the 2018 American Society of Clinical Oncology/College of American Pathologists guidelines. HER2 status was first determined by immunohistochemistry (IHC), and patients with IHC 3 + tumors were diagnosed as HER2 positive; for patients with HER2 IHC 2 + tumors, HER2 was identified as positive by FISH. The detection of ER and PR was based on the 2020 American Society of Clinical Oncology/College of American Pathologists guidelines, and tumors were classified as hormone receptor (HR) positive if ≥1% of tumor cells stained for ER and/or PR.

**Figure 1.**
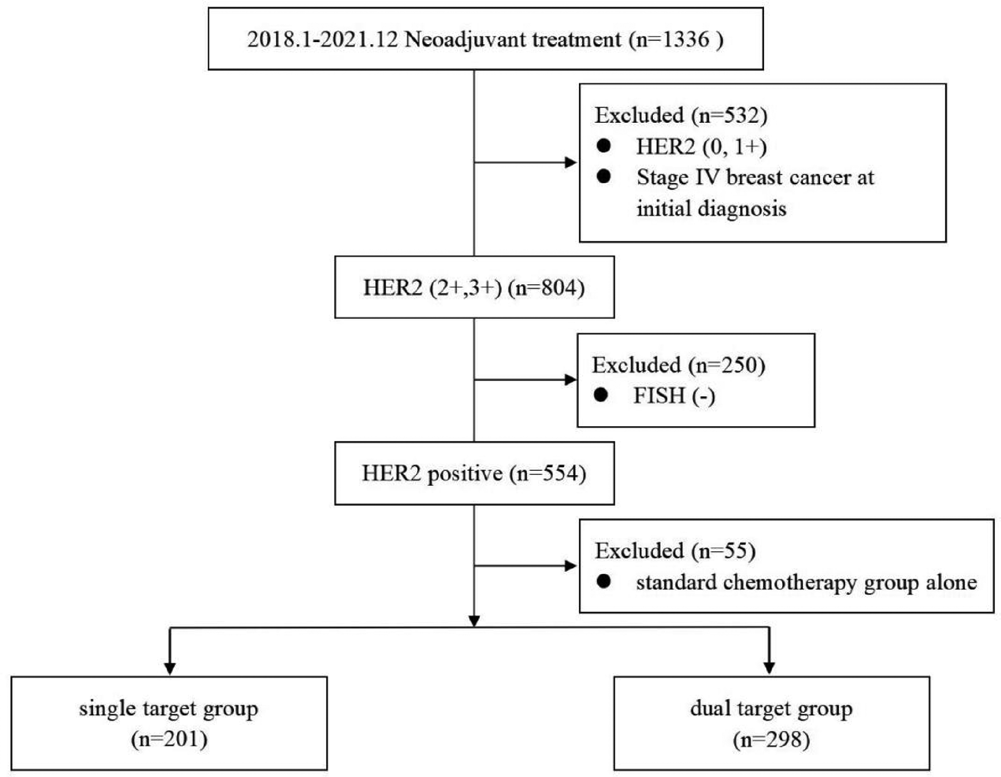
Flow chart of case screening.

This study was approved by the Ethics Committee of the Fourth Hospital of Hebei Medical University (Ethics number: 2020048) (Fig. 1 and Table 1).

**Table 1:** Basic characteristics of 499 patients with HER2 positive breast cancer (N [%]).

Clinical characteristics	Single target group N = 201	Dual target group N = 298	*χ* ^²^	*P*
Age			0.132	.716
<50	91 (45.27)	130 (43.62)		
≥50	110 (54.73)	168 (56.38)		
Menopause			2.631	.105
Yes	89 (44.28)	154 (51.68)		
No	112 (55.72)	144 (48.32)		
Clinical stage			4.912	.555
IIA	25 (12.44)	31 (10.40)		
IIB	62 (30.85)	103 (34.56)		
IIIA	35 (17.41)	63 (21.14)		
IIIB	27 (13.43)	34 (11.41)		
IIIC	52 (25.87)	67 (22.48)		
T stage			1.149	.765
T1	21 (10.45)	23 (7.72)		
T2	120 (59.70)	185 (62.08)		
T3	24 (11.94)	37 (12.42)		
T4	36 (17.91)	53 (17.79)		
N stage			2.311	.510
N0	17 (8.46)	19 (6.38)		
N1	95 (46.77)	154 (51.67)		
N2	36 (17.91)	59 (19.79)		
N3	53 (26.37)	66 (22.15)		
Hormone receptor			0.086	.769
HR positive	118 (58.71)	171 (57.38)		
HR negative	83 (41.29)	127 (42.62)		
HER2 status			2.925	.087
HER2 3+	184 (91.54)	258 (86.58)		
HER2 2 + FISH+	17 (8.46)	40 (13.42)		
Ki-67			2.868	.090
≤20	52 (25.87)	58 (19.46)		
>20	149 (74.13)	240 (80.54)		

HER2 = human epidermal growth factor receptor 2.

### 2.2. Statistical analysis

The primary endpoint was the HER2-negative conversion rate, defined as among HER2-positive breast cancer after neoadjuvant treatment that did not reach pCR (non-pCR), there was the proportion of HER2 expression turned negative. Secondary study measures were pathologic complete response (pCR), defined as noninvasive carcinoma in the primary breast lesion (possibly ductal carcinoma in situ) and lymph nodes in the negative area (ypT0 or Tis N0M0), or residual cancer burden grade 0. Disease free survival (DFS) is the time from breast cancer diagnosis to the recurrence of ipsilateral local or regional invasive breast cancer, distant metastasis, noninvasive breast cancer, or death from any cause. Overall survival (OS) was defined as the time between primary diagnosis and death from any cause. The survival time was calculated from the time of pathological diagnosis. The end date of follow-up was October 14, 2022, and the end event was the death of the patient. SPSS 26.0 was used for statistical processing. The number of cases (%) was expressed by *χ*^2^ test, *Fisher* exact test, and Log-Rank test. Test level α = 0.05 (dual-tailed).

## 3. Results

### 3.1. Clinical efficacy of HER2-positive breast cancer

After neoadjuvant treatment, 255 patients achieved pCR, with a total PCR rate of 51.10%. The pCR rates of HR- group and HR + group were 64.76% (136/210) and 41.18% (119/289), respectively, and the difference was statistically significant (*χ*^2^ *=* 27.075, *P* < .001). The pCR rates of the dual target group and single target group were 60.07% (179/298) and 37.81% (76/201), respectively, and the difference was statistically significant (*χ*^2^ = 23.795, *P* < .001). In the HER2-positive HR-negative group, 136 cases (64.76%) achieved pCR, of which 96 cases (75.59%) in the dual target group and 40 cases (48.19%) in the single target group, and the difference was statistically significant (*χ*^2^ = 16.511, *P* < .001). In the HER2-positive HR-positive group, 119 patients (41.18%) achieved pCR, including 83 patients (48.54%) in the dual-target group and 36 patients (30.51%) in the single-target group, and the difference was statistically significant (*χ*^2^ = 9.370, *P* = .002). Among the 255 patients who achieved pCR, the pCR rate of the HER2 3 + group was 56.11% (248/442), and the pCR rate of the HER2 2 + FISH (+) group was 12.28% (7/57), and the difference was statistically significant (*χ*^2^ = 38.812, *P* < .001) (Table 2).

**Table 2:** Efficacy of neoadjuvant treatment in 499 cases of HER2-positive breast cancer (N [%]).

	N	pCR	non-pCR	*χ* ^²^	*P*
Hormone receptor (n = 499)				27.075	<.001
HR-negative	210	136 (64.76)	74 (35.24)		
HR-positive	289	119 (41.18)	170 (58.82)		
Neoadjuvant regimen (n = 499)				23.795	<.001
Dual target group	298	179 (60.07)	119 (39.93)		
Single target group	201	76 (37.81)	125 (62.19)		
HER2-positive HR-negative (n = 210)				16.511	<.001
Dual target group	127	96 (75.59)	31 (24.41)		
Single target group	83	40 (48.19)	43 (51.81)		
HER2-positive HR-positive (n = 289)				9.370	.002
Dual target group	171	83 (48.54)	88 (51.46)		
Single target group	118	36 (30.51)	82 (69.49)		
HER2 status (n = 499)				38.812	<.001
HER2 3+	442	248 (56.11)	194 (43.89)		
HER 2 + FISH +	57	7 (12.28)	50 (87.72)		

HER2 = human epidermal growth factor receptor 2, HR = hormone receptor, pCR = pathological complete response, non-pCR = non-pathological complete response.

### 3.2. Changes in HER2 expression after neoadjuvant treatment

A total of 244 patients with HER2-positive breast cancer did not achieve pCR after neoadjuvant treatment, including 119 patients in the dual target group and 125 patients in the single target group. There was a negative conversion rate of HER2 expression status after neoadjuvant treatment in patients without pCR, and the overall negative conversion rate was 13.11% (32/244). The negative conversion rates of HER2 expression in the dual target group and the single target group were 17.65% and 8.80%, respectively, and the difference was statistically significant (*χ*^2^ = 4.188, *P* = .041). In the HER2-positive HR-negative groups, the negative conversion rates of HER2 expression in the dual target group and the single target group were 12.90% and 7.00%, respectively, and the difference was not statistically significant (*P* = .394). In the HER2-positive HR-positive group, the negative conversion rates of HER2 expression in the dual target group and the single target group were 19.32% and 9.76%, respectively, and the difference was statistically significant (*P* < .001). Among 244 patients who did not achieve pCR, the negative conversion rate of HER2 3 + and HER2 2 + FISH(+) before neoadjuvant therapy was 6.19% (12/194) and 40% (20/50), respectively, with a significant difference (*P* <.001). (Table 3).

**Table 3:** Changes of HER2 expression before and after new adjuvant treatment in 244 non-pathological complete response (non-PCR) patients (N [%]).

	N	Positive to negative	Positive	*χ* ^²^	*P*
				4.188	.041
Dual target group	119	21 (17.65)	98 (82.35)		
Single target group	125	11 (8.80)	114 (91.20)		
HER2-positive HR-negative (n = 74)				0.727^a^	.394
Dual target group	31	4 (12.90)	27 (87.10)		
Single target group	43	3 (7.00)	40 (93.00)		
HER2-positive HR-positive (n = 170)				39.880*	<.001
Dual target group	88	17 (19.32)	71 (80.68)		
Single target group	82	8 (9.76)	74 (90.24)		
HER2 status				39.891	<.001
HER2 3+	194	12 (6.19)	182 (93.81)		
HER 2 + FISH +	50	20 (40.00)	30 (60.00)		

HER2 = human epidermal growth factor receptor 2, HR = hormone receptor.

* Fisher exact probability test.

### 3.3. Immunohistochemistry of HER2 after turning negative

Among the 32 patients with negative conversion, 7 cases were the HER2-positive HR-negative before neoadjuvant treatment, 6 cases (18.75%) became triple negative breast cancer (TNBC) and 1 case (3.125%) became Luminal B after neoadjuvant treatment, and 25 cases were the HER2-positive HR-positive before neoadjuvant treatment. After neoadjuvant treatment turned negative, 1 case (3.125%) became TNBC, 2 cases (6.25%) were Luminal A and 22 cases (68.75%) were Luminal B. After neoadjuvant therapy, Ki-67 expression decreased in 24 cases (75%), remained unchanged in 3 cases (9.375%), and increased in 5 cases (15.625%).

### 3.4. Prognosis condition

**Figure 2.**
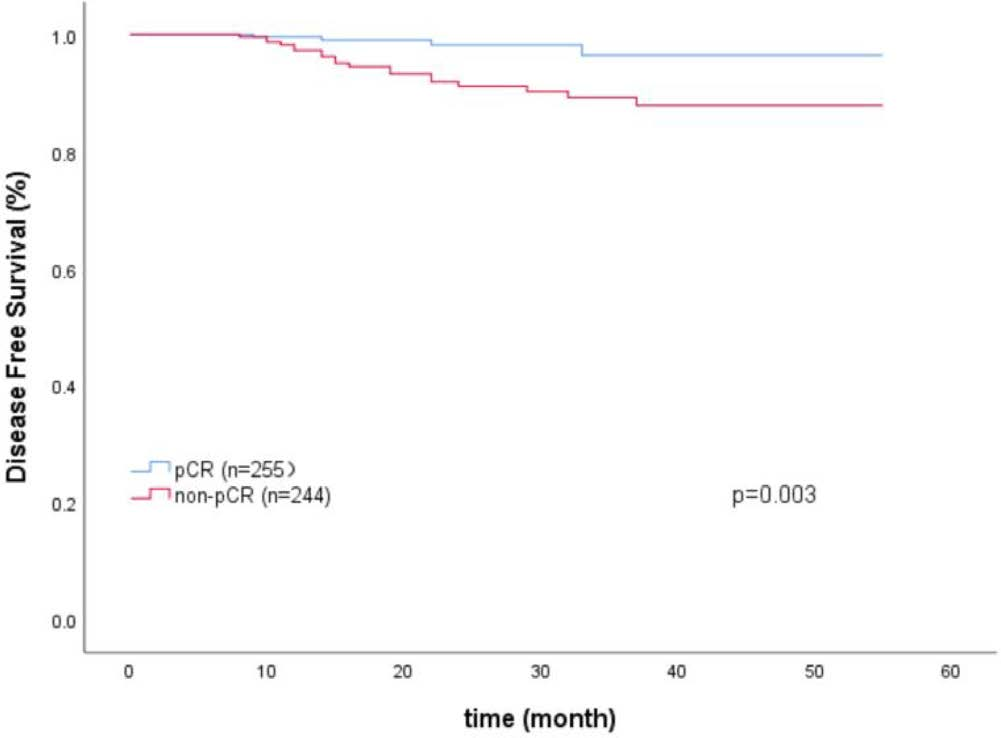
Kaplan–Meier disease free survival curves according pathologic complete response (pCR) status after neoadjuvant treatment.

**Figure 3.**
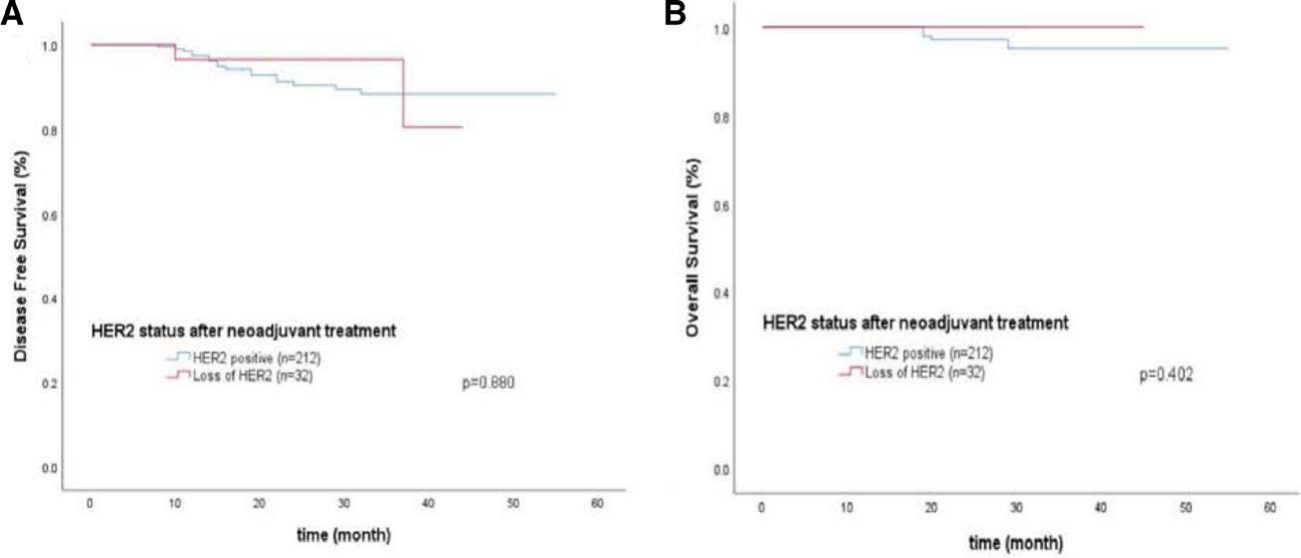
Kaplan–Meier disease free survival curves (A) and overall survival curves (B) were plotted according to the HER2 status of residual tumors in Non-pathological complete response (non-pCR) patients after neoadjuvant targeted treatment.

Among the 499 HER2-positive patients, the follow-up data were complete, and the follow-up time ranged from 7 to 55 months, with a median follow-up time of 22 months. All patients continued targeted therapy for 1 year after surgery, and endocrine therapy and radiotherapy were supplemented according to the surgical method, postoperative pathological results, lymph node metastasis and postoperative HR status. Among the 255 patients with pCR, 4 (0.40%) had recurrence or metastasis, and 2 (0.40%) died. Among 244 non-pCR patients, 19 patients (2.61%) had recurrence or metastasis, and 6 patients (1.20%) died of brain metastasis or bone metastasis. The DFS of the pCR group was 98.43% (251/255), which was significantly higher than that of the non-pCR group 92.21% (225/244), Log-Rank test, *χ*^2^ = 8.536, *P* = .003. However, there was no significant difference in OS between the 2 groups (99.22% vs 97.54%), Log-Rank test, *χ*^2^ = 1.248, *P* = .264. Of the 32 patients who were HER2 negative after neoadjuvant treatment, 2 (0.20%) had recurrence and metastasis. The DFS of the HER2-negative group was 93.75% (30/32), which was not significantly different from that of the HER2-nonnegative group 91.98% (195/212) (Log-Rank test, *χ*^2^ = 0.023, *P* = .880). However, there was no significant difference in OS between the 2 groups (100% vs 97.17%), Log-Rank test, *χ*^2^ = 0.703, *P* = .402 (Figs. 2 and 3).

## 4. Discussion

Tumor heterogeneity refers to the differences in the characteristics of tumor cells caused by the existence of different genotype cells in the same tumor.^[7]^ HER2-positive breast cancer has poor differentiation ability and strong tumor invasiveness, which can increase the risk of breast cancer recurrence and metastasis, and has significant heterogeneity.^[8]^ For HER2-positive breast cancer, neoadjuvant targeted treatment can greatly improve the prognosis of patients and prolong their survival.^[9,10]^ However, breast cancer with HER2 heterogeneity has a poor prognosis and is not sensitive to anti-HER2 treatment.^[11]^ The pCR rate was 51.10% (255/499) in 499 patients with HER2-positive breast cancer after neoadjuvant treatment. Among, pCR was more easily obtained in HR−/HER2-positive group after neoadjuvant therapy than in HR+/HER2-positive group. Moreover, the pCR rate of the HER2 3 + group (56.11%) was higher than that of the HER2 2 + FISH (+) group (12.28%), which was consistent with previous reports.^[12,13]^ It indicates that HR status and HER2 protein expression level also have important effects on pCR.

Previous studies have shown that HER2 expression becomes negative in patients with HER2-positive breast cancer after neoadjuvant treatment, and the negative conversion rate of HER2 expression ranges from 9.4% to 40%. Mittendorf et al^[14]^ found that among the patients who did not achieve pCR after neoadjuvant trastuzumab treatment, there were 25 patients with sufficient residual cancer tissue to retest HER2 expression status, and 8 (32%) of them were negative. Guarneri et al^[15]^ found that HER2 expression turned negative in 19 of 69 residual cancer tissues after neoadjuvant treatment, with a negative conversion rate of 27.5%. Yoshida et al^[16]^ found that 33 (33%) of 99 patients with HER2-positive breast cancer without pCR turned negative, among whom trastuzumab neoadjuvant treatment had the highest negative conversion rate of 70% (23/33). Wang et al^[17]^ found that anti-HER2 neoadjuvant targeted treatment is more likely to become turn negative. The negative conversion rate of the PCH (paclitaxel, carboplatin, trastuzumab) regimen was 19.8%, while that of the PC (paclitaxel, carboplatin) regimen was 9.4%, and the difference was statistically significant. This study showed that in 499 patients with HER2-positive breast cancer after neoadjuvant treatment, 32 of 244 cases (13.11%) that did not achieve pCR turned HER2 negative. Among them, 21 cases (17.65%) in the dual target group turned HER2 expression negative, which was higher than 11 cases (8.80%) in the single target group. In the HER2-positive HR-negative group and HER2-positive HR-positive group, the negative conversion rate of HER2 expression status in the dual target group was also higher than that in the single target group. Especially in the HER2-positive HR-positive group, the negative conversion rate of the dual target group was as high as 19.32%, suggesting that patients have a higher negative conversion rate after dual target neoadjuvant treatment.

Previous studies have shown that neoadjuvant treatment with trastuzumab plus chemotherapy results in a higher rate of HER2-negative conversion than neoadjuvant treatment with chemotherapy alone. However, in this study, the HER2-negative conversion rate of dual target neoadjuvant treatment was higher than that of the single-target group. The reason for this inconsistency is that the previous studies did not use dual target neoadjuvant treatment as a comparison. A second possibility is the use of different criteria for HER2 testing of residual tumors. The mechanism of HER2 reversion to negative status after neoadjuvant treatment is unclear. Among 244 patients who did not achieve pCR, the negative conversion rates of HER2 3 + and HER2 2 + FISH (+) before neoadjuvant treatment were 6.19% (12/194) and 40% (20/50), respectively (*P* < .001). Some studies have shown that HER2 2 + heterogeneity is more obvious.^[18]^ HER2-negative conversion after neoadjuvant treatment may be related to tumor heterogeneity and drug resistance, suggesting that there is HER2 heterogeneity within the tumor, HER2-positive tumor cells are effective to chemotherapy and targeted treatment, residual HER2-negative tumor cells, or HER2 positive tumor cells are resistant to trastuzumab.

Among 32 HER2-positive patients, 7 (21.875%) became TNBC after neoadjuvant therapy, 2 (6.25%) became Luminal A and 23 (3.125%) became Luminal B. Braso-Maristany et al^[19]^, in HER2 overexpression, showed that trastuzumab and lapatinib treatment can induce low proliferation of Luminal A type. The subtype switch from HER2 overexpression to Luminal A may provide an opportunity for treatment with agents known to be active in Luminal A, such as endocrine treatment or CDK4/6 inhibitors. Therefore, when HER2 becomes negative and becomes a different molecular subtype, it may be necessary to add new treatment options to push HER2 targeted treatment to a higher level.

In this study, 32 patients with HER2 positive turned negative after neoadjuvant treatment, and the immunohistochemical indexes of these 32 patients before and after neoadjuvant treatment were observed, it was found that Ki-67 generally showed a downwards trend, of which 24 cases decreased (75%), 3 cases remained unchanged (9.375%), and 5 cases increased (15.625%). Gahlaut et al^[20]^ found that Ki-67 changes after neoadjuvant treatment were statistically significant. Studies have shown that Ki-67 expression is significantly decreased after neoadjuvant treatment, and high Ki-67 expression in residual tumors is associated with poor DFS and OS. Therefore, decreased Ki-67 expression after neoadjuvant treatment in breast cancer patients indicates a better prognosis.^[21]^

A total of 499 HER2-positive patients were followed up, and it was found that patients who achieved pCR had a better prognosis, which was consistent with previous studies.^[11]^ Two of 32 patients (0.20%) with HER2 negative conversion had recurrence and metastasis, and there were no deaths. Its low recurrence and metastasis rates show a good prognosis trend, which may be related to the continued targeted treatment and other intensive treatments after surgery. Some studies have shown that HER2 positive conversion after neoadjuvant treatment is not associated with 5-year RFS or OS, and these patients are recommended to continue to complete targeted adjuvant treatment.^[22]^ In addition, the KATHERINE study showed the efficacy of T-DM1 in the treatment of HER2-negative residual invasive disease. Among 70/845 (8.3%) patients with HER2-negative residual disease reviewed at surgery, 11 of 42 (26.2%) patients treated with trastuzumab had IDFS events. However, there was no IDFS event in the 28 patients treated with T-DM1, indicating that T-DM1 treatment should not be discontinued in this patient population.^[23]^

However, the sample size of this study is small and has certain limitations. In the future, the sample size should be expanded, and the heterogeneity of HER2 and the potential impact of targeted drugs on HER2 biology should be further understood, as well as the resistance mechanism of anti-HER2 treatment. In addition, the optimal treatment strategy for patients with HER2 turning negative still needs to be explored, but at present, it is still recommended to continue anti-HER2 treatment clinically.

## Author contributions

Writing – original draft: Xiaofei Ren, Xiangmei Zhang.

Data curation: Xiangmin Ma, Chao Yang, Jingping Li, Beichen Liu, Chao Shi.

Writing – review & editing: Yunjiang Liu.
